# A Randomized Controlled Trial Comparing Suture-Fixation Mucopexy and Doppler-Guided Hemorrhoidal Artery Ligation in Patients with Grade III Hemorrhoids

**DOI:** 10.1155/2016/8143703

**Published:** 2016-03-15

**Authors:** Min Zhai, Yong-An Zhang, Zhen-Yi Wang, Jian-Hua Sun, Jie Wen, Qi Zhang, Jin-De Li, Yi-Zheng Wu, Feng Zhou, Hui-Lei Xu

**Affiliations:** ^1^Department of Anorectal Surgery, The TCM Hospital of Pu Dong New District, Shanghai 201299, China; ^2^Yueyang Hospital of Integrated Chinese and Western Medicine, Shanghai University of Traditional Chinese Medicine, Shanghai 200437, China

## Abstract

*Background*. We aimed to evaluate the effectiveness of a suture-fixation mucopexy procedure by comparing with Doppler-guided hemorrhoidal artery ligation (DGHAL) in the management of patients with grade III hemorrhoids.* Methods. *This was a randomized controlled trial. One hundred patients with grade III hemorrhoids were randomly assigned to receive suture-fixation mucopexy (*n* = 50) or DGHAL (*n* = 50). Outcome assessments were performed at 2 weeks, 12 months, and 24 months. Assessments included resolution of clinical symptoms, postoperative complications, duration of hospitalization, and total costs.* Results*. At 2 weeks, one (2%) patient in suture-fixation group and four (8%) patients in DGHAL group had persistent prolapsing hemorrhoids. Postoperative bleeding was observed in two patients (4%) in suture-fixation group and one patient in DGHAL group. There was no significant difference in short-term recurrence between groups. Postoperative complications and duration of hospitalization were comparable between the two groups. Rates of recurrence of prolapse or bleeding at 12 months did not differ between groups. However, recurrence of prolapse at 24 months was significantly more common in DGHAL group (19.0% versus 2.3%, *p* = 0.030).* Conclusions*. Compared with DGHAL, the suture-fixation mucopexy technique had comparable short-term outcomes and favorable long-term outcomes.

## 1. Introduction

Hemorrhoids are the most common proctological diseases and affect between 4.4 and 36.4% of the general population [1]. It is a condition with a variety of symptoms and a spectrum of severity. Although the majority of patients with grade I and II hemorrhoids can be effectively managed with conservative treatment, surgical intervention is required for patients with advanced stages of hemorrhoids [2]. While hemorrhoidectomy remains the gold-standard approach for grade IV hemorrhoids, several minimally invasive treatment options, such as Doppler-guided hemorrhoidal artery ligation (DGHAL), have been introduced for the management of grade III hemorrhoids, aiming at overcoming the disadvantages associated with hemorrhoidectomy, including severe postoperative pain and complications such as anal stricture [3–5]. DGHAL was introduced in 1995 by Morinaga et al. [4]. This method requires the use of a proctoscope with a Doppler transducer attached to detect the location and depth of arterial structures. Studies of DGHAL have shown encouraging short-term results in terms of postoperative morbidity for symptomatic hemorrhoids [6, 7].

In this study, we introduced a simple mucopexy procedure by suture-fixation of anal cushion to manage prolapsing hemorrhoids. It is developed based on Thompson's theory that hemorrhoids are the results of the sliding anal lining mucosa of the anal cushion [8]. The procedure involves stitches which transfix the base of the protruding hemorrhoids, followed by sutures on the entire protruding hemorrhoids, to restore fixation of the cushions to its original position, thus controlling the prolapse. In this randomized controlled trial with two-year follow-up, by comparing with DGHAL, we aimed to evaluate the effectiveness of this suture-fixation procedure with regard to resolution of hemorrhoid symptoms, duration and costs of operation, postoperative morbidity and complications, and long-term recurrence, in the management of patients with grade III hemorrhoids.

## 2. Patients and Methods

### 2.1. Study Design and Patients

This is a randomized controlled clinical trial. Consecutive patients aged 18 years or older with symptomatic grade III internal hemorrhoids who required surgery were enrolled at our hospital during March 2010 and May 2011. Diagnosis of hemorrhoids was confirmed by physical examination and anoscopy or proctoscopy and severity of hemorrhoids was according to the Goligher classification [9]. Exclusion criteria included surgical history for hemorrhoids within three years, previous major surgery to the rectum, firm and fibrotic external irreducible hemorrhoids, thrombosed hemorrhoids, presence of other anal disorders including abscesses and rectal or anal cancer, history of inflammatory bowel disease, pregnancy, and inability to give informed consent. Patients were randomly assigned to receive one of the two procedures (suture-fixation group and DGHAL group). Randomization was done by computer-generated random numbers and numbered and sealed envelopes, which were opened in the operating room before the surgery. Patients and the surgical team were blinded to the assigned procedures until the surgery. All surgical procedures were conducted by the same operating team led by the same surgeon. The study protocol was reviewed and approved by the ethics committee of the TCM Hospital of Pu Dong New District, Shanghai. All study procedures were performed in accordance with the Declaration of Helsinki. All participants provided written informed consent.

**Figure 1:**
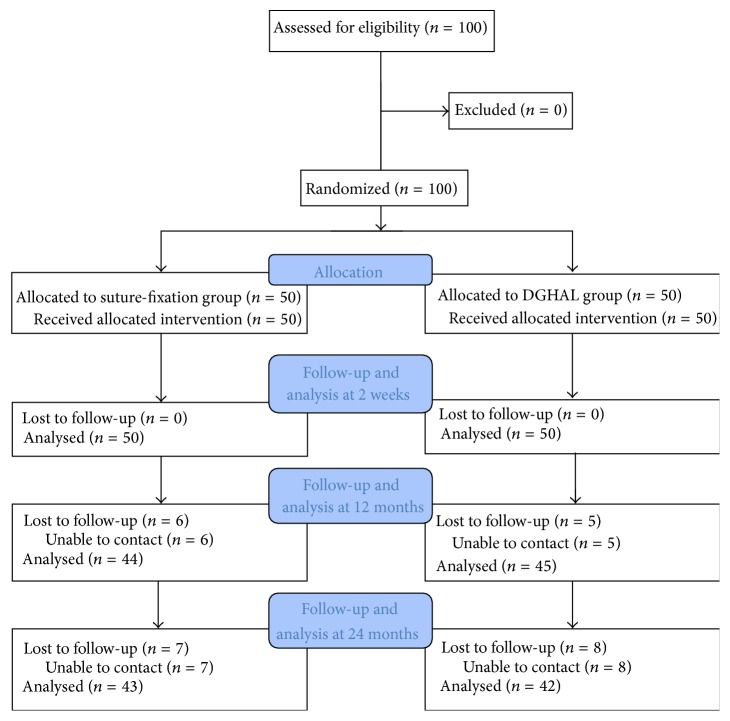
CONSORT flow chart of study enrollment. DGHAL: Doppler-guided hemorrhoidal artery ligation.

A total of 100 patients (50 patients for each group) were enrolled for the trial.  Figure 1 shows the enrollment flow for this study.  Table 1 shows the baseline characteristics of the study subjects. The two groups were comparable with respect to age, gender, disease duration, and the number of hemorrhoids.

### 2.2. Surgical Procedures

#### 2.2.1. Suture-Fixation Group

**Figure 2:**
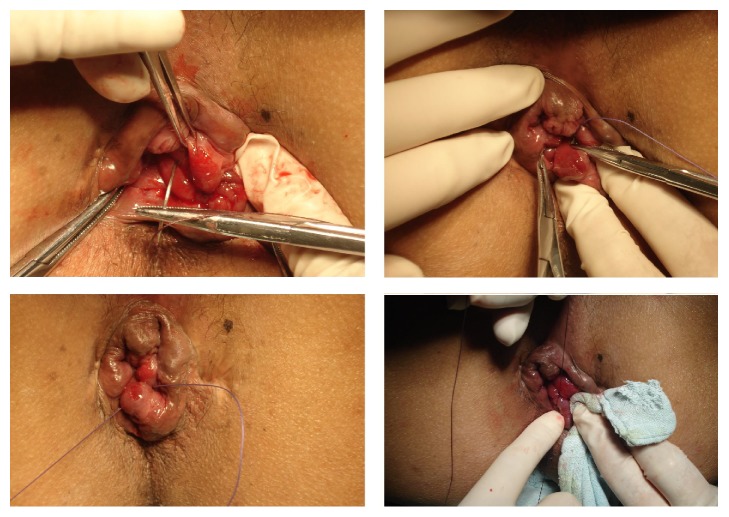
Procedure of suture-fixation mucopexy.

The procedure was performed under spinal or general anesthesia with the patients in the lithotomy position. After cleaning the perineal skin region and covering the patient with sterile draping, the anal canal was gently dilated by the passage of two fingers. An anoscope was inserted to examine the sites of hemorrhoidal cushions. After retracting the anoscope, a suture anoscope (diameter of 3 cm, length of 8 cm, JINTAN, Jiangsu, China) was introduced through the anal canal. With clear exposure of the dentate line and displaced rectal mucosa, the prolapsing hemorrhoids were pushed back into the anal canal. Two continuous sutures were performed using a UR-6, 26 mm, 5/8c vicryl needle, and an absorbable 0 chromic catgut (Ethicon, US) at 2 mm above the dentate line and the stitches were passed through the mucous membrane and the muscular layer of the rectal wall beneath the prolapsing hemorrhoids. This was followed by 2–4 sutures ascending cephalically, each with 2 mm distance, depending on the degree of prolapse of the hemorrhoids and the displacement of rectal mucosa (Figure 2). The procedure was performed for all hemorrhoids seen, usually towards 3, 7, and 11 o'clock. Any bleeding was stopped by simple pressure for a short while. A Vaseline gauze was inserted into the anal canal after the procedure.

#### 2.2.2. DGHAL Group

The procedure was performed under spinal or general anesthesia with the patients in the lithotomy position. The DGHAL device (HAL-Doppler II, AMI, Feldkirch, Austria) consists of a specially designed proctoscope equipped with a Doppler probe and a light source. After cleaning the perineal skin region and covering the patient with sterile draping, the anal canal was gently dilated to a width of two fingers and the proctoscope was inserted with the dentate line and displaced rectal mucosa was clearly exposed and the prolapsing hemorrhoids were pushed back into the anal canal. A Doppler probe was then inserted into the anal canal and placed about 2-3 cm above the dentate line, starting at 6 o'clock and going clockwise, to identify the branch of the upper rectal artery. Once accurate detection was confirmed via Doppler sound and the vessel depth was displayed on the apparatus screen, a figure-of-eight suture was conducted using a UR-6, 26 mm, 5/8c vicryl needle, and an absorbable 2-0 chromic catgut (Ethicon, US) where the artery had been identified. After accurate ligation had been confirmed via loss of the Doppler vein signal, it was firmly ligated using a pusher. The above procedure was performed on six sites (towards 1, 3, 5, 7, 9, and 11 o'clock) to ensure that all arteries were located and ligated, and no more prolapsing hemorrhoids or artery signal was found. A Vaseline gauze was inserted into the anal canal after the procedure.

### 2.3. Postoperative Management and Assessments

Patients were instructed to be on bed rest on the day of the procedure. A two-day course of antibiotics and stool softener were prescribed after the procedure. Hospital discharge was issued if the patient did not complain of bleeding or urinary retention and if the patient was ambulatory for daily activities. All outcome assessments were performed by an independent blinded assessor at 2 weeks, 12 months, and 24 months after the procedures. At 2 weeks, outcome assessments included (1) effectiveness of the procedures, which was classified as clinical symptoms completely resolved (absence of clinical symptoms, significant shrinkage, or disappearance of hemorrhoids), partially resolved (improved clinical symptoms, mild shrinkage of hemorrhoids), and persistent (no improvement in clinical symptoms), (2) postoperative recurrence of prolapsing hemorrhoids and bleeding, (3) duration of hospitalization, (4) total costs of the procedure, (5) and postoperative complications, including bleeding, anal discomfort, urinary retention, anal stricture, and fecal incontinence. In addition, assessment of postoperative pain was conducted at 1, 2, and 3 days after the procedures using a 10 cm linear visual analogue scale (VAS) in which 0 corresponded to “no pain” and 10 to “maximum pain.” At 3 months after the procedures, fecal incontinence was assessed using the Wexner Incontinence Grading Scale [10]. The total score is derived from the sum of the numerical values assigned to the frequency of occurrence (scored 0–4) in each of four categories: type of incontinence (solid, liquid, and gas), pad use, and lifestyle alteration. Total score ranges from 0 (perfect continence) to 20 (complete incontinence). Long-term telephone follow-up assessments were conducted at 12 and 24 months, including incidence of recurrence (prolapsing hemorrhoids or bleeding) and patients' satisfaction (classified as excellent, good, fair, and poor).

### 2.4. Statistical Analyses

Statistical analyses were performed using SPSS (version 17.0, SPSS Inc., Chicago, IL). Results were expressed as either mean and standard deviation (SD) or frequency (percentage). Between-group comparisons were performed using Student's *t*-test (normally distributed data) or Mann-Whitney *U* test (skew data) for continuous variables or Chi-square test for categorical variables. All hypotheses were two-tailed with *p* < 0.05 considered as statistically significant. Sample size was calculated to detect a significant difference in success rate defined as proportion of patients with symptoms completely resolved after the procedure. In an a priori Fisher exact sample size calculation, 50 patients in each group would be needed to detect a 20% difference in success rate between the two groups, assuming a power of 80%, a type I error probability of 5%, and an allocation ratio of 1.

## 3. Results

### 3.1. Short-Term Outcomes

After the procedures, in suture-fixation group, effectiveness of the procedures was classified as symptoms completely resolved, partially resolved, and persistent in 43 (86%), 6 (12%), and 1 (2%) patients, respectively. The corresponding figures in DGHAL group were 35 (70%), 11 (22%), and 4 (8%), respectively. There was significant between-group difference in percentage of resolution of clinical symptoms (*p* = 0.048).

Postoperative bleeding was observed in two patients (4%) in suture-fixation group. One patient developed significant bleeding two days after the procedure which was successfully managed with simple ligation under local anesthesia. The other patient developed persistent oozing of blood which was successfully managed with intravenous use of hemocoagulase. Postoperative oozing of blood was observed in one case (2%) in DGHAL group which was managed by simple mechanical compression. One (2%) patient in suture-fixation group and four (8%) patients in DGHAL group had persistent prolapsing hemorrhoids which were further managed by sclerotherapy. There was no significant between-group difference in the percentage of postoperative bleeding or prolapsing (Table 2).

There was no adverse event during the procedures. After the procedures, in suture-fixation group, anal discomfort and urinary retention were presented in 15 (30%) patients and 7 (14%) patients, respectively (Table 2). The corresponding figures in DGHAL group were 7 (14%) and 5 (10%), respectively, with no significant between-group difference. No patient developed anal stricture or fecal incontinence. There were no significant differences in duration of hospitalization (7.04 ± 1.78 versus 5.3 ± 1.25 days, *p* = 0.510) and total costs of the procedures were significantly lower in suture-fixation group than in DGHAL group (3,138 ± 552 versus 4,020 ± 673 Chinese Yuen, *p* < 0.001).

Table 2 shows the pain VAS scores 1, 2, and 3 days after the procedures. At each time point, pain score did not differ significantly between suture-fixation group and DGHAL group.  Table 3 shows the total scores of the Wexner Incontinence Grading Scale 3 months after the procedures. There was no significant between-group difference in the total Wexner score.

### 3.2. Long-Term Outcomes

Follow-up at 12 months was completed in 89 patients and follow-up at 24 months in 85 patients (Table 4). At 12 months, four patients in DGHAL group had recurrent prolapsing hemorrhoids, two of which were managed with hemorrhoidectomy and the other two refused further management. No patient in suture-fixation group had recurrent prolapsing hemorrhoids. Occasional bleeding that was managed by conservative treatments was reported in two patients in suture-fixation group and one patient in DGHAL group. The percentage of recurrent prolapsing hemorrhoids or bleeding did not differ between the two groups. At 24 months, the percentage of recurrent prolapsing hemorrhoids (7% versus 23.8%, *p* = 0.03) or bleeding (2.3% versus 19%, *p* = 0.03) was significantly lower in suture-fixation group than in DGHAL group. At both 12 and 24 months, ratings of patients' satisfaction were significantly higher (rated as excellent or good) in suture-fixation group than in DGHAL group.

## 4. Discussion

In this study, we evaluated the effectiveness of suture-fixation mucopexy procedures in the treatment of grade III hemorrhoids compared with currently widely used DGHAL. In the comparisons in short-term postoperative outcomes, with respect to resolution of symptoms, pain, complications, and fecal incontinence, there was no significant difference between the two treatments. Total costs of the treatment were significantly lower for suture-fixation mucopexy. Long-term outcomes at 12 months, with respect to recurrence of symptoms, were also comparable between the two treatments. However, long-term outcomes at 24 months were in favor of suture-fixation mucopexy, which showed significantly lower incidence of recurrent prolapsing hemorrhoids and bleeding. Our results supported that this simpler, easy-to-learn, and minimally invasive technique could be a potential alternative to current treatment options in the management of symptomatic hemorrhoids.

The anal cushions are a normal component of the anal canal that consist of vascular, connective tissue, and elastic fibers and collagen, lined by cylindrical epithelium [11]. Although the purpose of anal cushions is not completely understood, they appear to play an important role in sensing fullness and pressure and in perceiving anal contents. They may also support anal closure, facilitate continence, and help protect the anal sphincter from injury during defecation [12]. Therefore, treatment principles of hemorrhoids are to minimize clinical symptoms while preserving the natural function of the anal cushions, in order to shorten the time to return to daily activities [13]. Traditional excisional hemorrhoidectomy is the most appropriate treatment for patients with grade IV hemorrhoids. The Milligan-Morgan (open) and Ferguson (closed) hemorrhoidectomy are the most commonly used surgical techniques. However, hemorrhoidectomy is notable to be associated with intense and prolonged postoperative pain [14]. Other complications associated with hemorrhoidectomy also occur with significant frequency. These include urinary retention (2–36%), bleeding (0.03–6%), anal stenosis (0–6%), and infection (0.5–5.5%) [14]. Sphincter defect or incontinence has been reported in up to 12% of patients after the surgery [15, 16]. To overcome postoperative pain, several new surgical modalities have been developed, such as laser hemorrhoidectomy, Harmonic Scalpel*™*, LigaSure*™*, Starion*™* sealing devices, and Starion Thermal Welding System. Hemorrhoidectomy with energy-based devices may cause less pain postoperatively than conventional excisional hemorrhoidectomy [17–20].

Several minimally invasive treatments, such as stapled hemorrhoidopexy and hemorrhoidal artery ligation (HAL), are proposed as an alternative to hemorrhoidectomy for the management of symptomatic hemorrhoids [21]. Stapled hemorrhoidopexy results in a stapled mucosa anastomosis in the rectum to reduce hemorrhoidal prolapse. A meta-analysis reported that stapled hemorrhoidectomy was less painful than conventional hemorrhoidectomy but also less effective, with recurrence rate of 7% compared with 2% in conventional hemorrhoidectomy in the medium term [22]. HAL involves a group of operations that ligate the blood vessels presumed to supply the hemorrhoid. DGHAL is the originally described technique. Because DGHAL does not involve tissue excision, it is associated with markedly reduced postoperative pain when compared with hemorrhoidectomy [23, 24]. A systematic review of 28 studies and a total of 2,904 patients reported an overall recurrence rate of 17.5%, a postoperative bleeding rate of 5%, and a reintervention rate of 6.4% [25]. The recurrence rate at five years has been reported to be 28% [6]. In order to secure the hemorrhoidal prolapse into the anal canal and to improve the efficacy, DGHAL can be modified by including a rectoanal repair or mucopexy (HAL-RAR) [26]. One-year recurrence rate for HAL-RAR has been reported to range between 11% and 14.4% [26, 27] and one study reported recurrence rate at 36 months as 9% [28]. The role of DGHAL has been questioned by recent randomized controlled studies showing that the rate of complications or recurrence or changes in vascular anatomy of the anal canal did not differ significantly between mucopexy plus DGHAL and non-Doppler-guided mucopexy [29, 30]. It is difficult to achieve complete and sustained loss of Doppler vein signal in DGHAL alone, which may explain the difficulty to reduce prolapse. This is supported by anatomical studies by Aigner et al. [31, 32]. Their studies showed branches of the superior rectal artery coursing in outer layers of the rectal wall and entering the rectal wall above the levator ani muscle to supply the corpus cavernosum recti [31]. While ligation of the main trunk of the superior rectal artery is possible with DGHAL, continuous hyperplasia of the branches of the superior rectal artery may be responsible for the persistent hemorrhoids and remarkable recurrent rate [32].

The sliding theory, popularized by Thomson, proposes that hemorrhoids are a result of sliding or displacement of anal lining mucosa of the anal cushions [8]. This theory is supported by the fact that hemorrhoids develop in patients with collagen fragmentation of the extracellular matrix and ligament of Treitz [33] and that mucosal prolapse usually proceeds hemorrhoidal bleeding [8]. Our suture-fixation technique is developed based on this theory, especially to address the increased laxity of the supportive structures that leads to prolapse. The stitches may also decrease the blood flow to hemorrhoid cushions, contributing to the shrinkage of hemorrhoids. Sutures are performed well above the dentate line to minimize postoperative pain. The procedure is minimally invasive, does not involve tissue excision, and is simple to perform. In our study, this procedure showed comparable short-term outcomes when compared with DGHAL, with respect to resolution of symptoms, pain, complications, fecal incontinence, and duration of hospitalization. Outcomes at 12 months were also comparable to those of DGHAL. However, costs of suture-fixation technique were significantly lower than those of DGHAL because it did not involve specially designed devices. It is noteworthy that long-term outcomes at 24 months were in favor of suture-fixation group, showing fewer recurrences. Higher ratings of patients' satisfaction at both 12 and 24 months were also observed in suture-fixation group. These results suggest that this suture-fixation technique has the potential as an effective and affordable treatment option for patients with symptomatic hemorrhoids.

Similar techniques of suture ligation of hemorrhoids by different approaches have been presented in some previous studies on a small scale [34–37]. Recently, Gupta et al. from India introduced a technique called “ligation and mucopexy of the hemorrhoids under vision” to tackle prolapsing hemorrhoids [29]. In this one-year randomized controlled trial compared with DGHAL with mucopexy (24 patients), patients treated with ligation and mucopexy (24 patients) had shorter operative time and lower postoperative pain at 6 weeks. Recurrence of hemorrhoids at one year did not differ between the two groups. Long-term follow-up for these techniques has shown shrunken and segmented hemorrhoids which are subsequently replaced by segmented fibrotic scar tissue that adhered firmly to the underlying structure [36].

Our study has several limitations. First, patients in control group received DGHAL alone, without mucopexy, which is the originally described technique for HAL. Our one-year recurrence rate for suture-fixation mucopexy appears to be lower than that reported for HAL-RAR [26, 27]. However, it would be interesting to compare the suture-fixation mucopexy technique with HAL-RAR to determine if this simple technique can yield comparable short-term and long-term outcomes. Second, long-term assessments included recurrence of symptoms. Long-term complication and reinterventional rate were not included. Third, the number of sutures was not recorded for each group for comparison. In suture-fixation group, it involved two continuous sutures followed by 2–4 sutures depending on the degree of prolapse. In DGHAL group, sutures were performed to ensure that all arteries were located and ligated. All study procedures were conducted by the same operating team led by the same surgeon. We would expect that the number of sutures should be likely related to the number of hemorrhoids of the patients, which was comparable between the two groups, as well as the degree of prolapse. The strength of our study was that it was a randomized controlled study on a relatively large cohort of patients and it had a longer follow-up.

In conclusion, compared with DGHAL, the suture-fixation mucopexy technique in our study had comparable short-term outcomes and favorable long-term outcomes. This technique has the potential as an effective and affordable treatment option for patients with grade III hemorrhoids.

## Figures and Tables

**Table 1:** Demographics and clinical characteristics of study subjects.

Variables	Suture-fixation group (*n* = 50)	DGHAL group (*n* = 50)	*p* value
Age, years	43.60 ± 14.97	50.56 ± 14.44	0.600
Disease duration, months	34.32 ± 15.19	35.82 ± 14.39	0.210
Male	23 (46%)	16 (32%)	0.150
Number of hemorrhoids	2.24 ± 0.92	2.86 ± 0.83	0.930

Results are mean ± SD or number (percentage). DGHAL: Doppler-guided hemorrhoidal artery ligation.

**Table 2:** Short-term recurrence of symptoms, postoperative complications, and postoperative pain score (visual analogue scale, 0–10) between the two groups.

Symptoms	Suture-fixation group (*n* = 50)	DGHAL group (*n* = 50)	*p* value
Recurrence			
Prolapse	1 (2%)	4 (8%)	0.359
Bleeding	2 (4%)	1 (2%)	1.000
Complications			
Anal discomfort	15 (30%)	7 (14%)	0.053
Urinary retention	7 (14%)	5 (10%)	0.675
Postoperative pain			
First day	3.4 ± 2.8	3.0 ± 3.0	0.069
Second day	1.7 ± 2.0	1.5 ± 1.9	0.074
Third day	1.0 ± 1.2	0.8 ± 1.4	0.093

Results are number (percentage) or mean ± SD. DGHAL: Doppler-guided hemorrhoidal artery ligation.

**Table 3:** Total scores of the Wexner Incontinence Grading Scale.

Scores	Suture-fixation group (*n* = 50)	DGHAL group (*n* = 50)	*p* value
0	41 (82%)	43 (86%)	0.600
1–5	6 (12%)	4 (8%)	
6–10	2 (4%)	3 (6%)	
11–15	1 (2%)	0	
16–20	0	0	

Results are number (percentage). DGHAL: Doppler-guided hemorrhoidal artery ligation.

**Table 4:** Long-term recurrence and patients' satisfaction.

Variables	12 months			24 months		
	Suture-fixation group (*n* = 44)	DGHAL group (*n* = 45)	*p* value	Suture-fixation group (*n* = 43)	DGHAL group (*n* = 42)	*p* value
Recurrence	2 (4.5%)	5 (11.1%)	0.450	3 (7.0%)	10 (23.8%)	**0.030**
Prolapse	0	4 (8.9%)	0.130	1 (2.3%)	8 (19.0%)	**0.030**
Bleeding	2 (4.5%)	1 (2.2%)	1.000	2 (4.7%)	2 (4.8%)	1.000
Patients' satisfaction						
Excellent	19 (43.2%)	10 (22.2%)	**0.012**	17 (39.5%)	10 (23.8%)	**0.042**
Good	23 (52.3%)	27 (60.0%)		22 (51.2%)	22 (52.4%)	
Fair	1 (2.3%)	5 (11.1%)		3 (7.0%)	6 (14.3%)	
Poor	1 (2.3%)	3 (6.7%)		1 (2.3%)	4 (9.5%)	

Results are number (percentage). Boldface indicates statistical significance. DGHAL: Doppler-guided hemorrhoidal artery ligation.

## References

[1] Loder P. B., Kamm M. A., Nicholls R. J., Phillips R. K. S. (1994). Haemorrhoids: pathology, pathophysiology and aetiology. *The British Journal of Surgery*.

[2] Altomare D. F., Giuratrabocchetta S. (2013). Conservative and surgical treatment of haemorrhoids. *Nature Reviews Gastroenterology and Hepatology*.

[3] Altomare D. F., Rinaldi M., Sallustio P. L., Martino P., De Fazio M., Memeo V. (2001). Long-term effects of stapled haemorrhoidectomy on internal anal function and sensitivity. *The British Journal of Surgery*.

[4] Morinaga K., Hasuda K., Ikeda T. (1995). A novel therapy for internal hemorrhoids: Ligation of the hemorrhoidal artery with a newly devised instrument (Moricorn) in conjunction with a Doppler flowmeter. *American Journal of Gastroenterology*.

[5] Tjandra J. J., Chan M. K. Y. (2007). Systematic review on the procedure for prolapse and hemorrhoids (stapled hemorrhoidopexy). *Diseases of the Colon and Rectum*.

[6] Avital S., Inbar R., Karin E., Greenberg R. (2012). Five-year follow-up of Doppler-guided hemorrhoidal artery ligation. *Techniques in Coloproctology*.

[7] Sohn N., Aronoff J. S., Cohen F. S., Weinstein M. A. (2001). Transanal hemorrhoidal dearterialization is an alternative to operative hemorrhoidectomy. *The American Journal of Surgery*.

[8] Thomson W. H. F. (1975). The nature of haemorrhoids. *The British Journal of Surgery*.

[9] Goligher J. C. (1980). Haemorrhoids or piles. *Surgery of the Anus, Rectum and Colon*.

[10] Jorge J. M. N., Wexner S. D. (1993). Etiology and management of fecal incontinence. *Diseases of the Colon & Rectum*.

[11] Jacobs D. (2014). Hemorrhoids. *The New England Journal of Medicine*.

[12] Schubert M. C., Sridhar S., Schade R. R., Wexner S. D. (2009). What every gastroenterologist needs to know about common anorectal disorders. *World Journal of Gastroenterology*.

[13] Rivadeneira D. E., Steele S. R., Ternent C., Chalasani S., Buie W. D., Rafferty J. L. (2011). Practice parameters for the management of hemorrhoids (revised 2010). *Diseases of the Colon and Rectum*.

[14] (2004). American Gastroenterological Association medical position statement: diagnosis and treatment of hemorrhoids. *Gastroenterology*.

[15] Felt-Bersma R. J. F., Van Baren R., Koorevaar M., Strijers R. L., Cuesta M. A. (1995). Unsuspected sphincter defects shown by anal endosonography after anorectal surgery. A prospective study. *Diseases of the Colon and Rectum*.

[16] Hiltunen K.-M., Matikainen M. (1992). Anal dilatation, lateral subcutaneous sphincterotomy and haemorrhoidectomy for the treatment of second and third degree haemorrhoids. A prospective randomized study. *International Surgery*.

[17] Chung C. C., Ha J. P., Tai Y. P., Tsang W. W., Li M. K. (2002). Double-blind, randomized trial comparing Harmonic Scalpel hemorrhoidectomy, bipolar scissors hemorrhoidectomy, and scissors excision: ligation technique. *Diseases of the Colon and Rectum*.

[18] Kwok S. Y., Chung C. C., Tsui K. K., Li M. K. W. (2005). A double-blind, randomized trial comparing ligasure*™* and Harmonic Scalpel*™* hemorrhoidectomy. *Diseases of the Colon and Rectum*.

[19] Wang J.-Y., Tsai H.-L., Chen F.-M. (2007). Prospective, randomized, controlled trial of Starion*™* vs. Ligasure*™* hemorrhoidectomy for prolapsed hemorrhoids. *Diseases of the Colon and Rectum*.

[20] Maloku H., Gashi Z., Lazovic R., Islami H., JunikuShkololli A. (2014). Laser hemorrhoidoplasty procedure vs open surgical hemorrhoidectomy: a trial comparing 2 treatments for hemorrhoids of third and fourth degree. *Acta Informatica Medica*.

[21] Hollingshead J. R., Phillips R. K. (2016). Haemorrhoids: modern diagnosis and treatment. *Postgraduate Medical Journal*.

[22] Jayaraman S., Colquhoun P. H., Malthaner R. A. (2006). Stapled versus conventional surgery for hemorrhoids. *Cochrane Database of Systematic Reviews*.

[23] Bursics A., Morvay K., Kupcsulik P., Flautner L. (2004). Comparison of early and 1-year follow-up results of conventional hemorrhoidectomy and hemorrhoid artery ligation: a randomized study. *International Journal of Colorectal Disease*.

[24] Wałȩga P., Scheyer M., Kenig J. (2008). Two-center experience in the treatment of hemorrhoidal disease using Doppler-guided hemorrhoidal artery ligation: functional results after 1-year follow-up. *Surgical Endoscopy*.

[25] Pucher P. H., Sodergren M. H., Lord A. C., Darzi A., Ziprin P. (2013). Clinical outcome following Doppler-guided haemorrhoidal artery ligation: a systematic review. *Colorectal Disease*.

[26] Satzinger U., Feil W., Glaser K. (2009). Recto Anal Repair (RAR): a viable new treatment option for high-grade hemorrhoids. One year results of a prospective study. *Pelviperineology*.

[27] Jeong W. J., Cho S. W., Noh K. T., Chung S. S. (2011). One year follow-up result of Doppler-guided hemorrhoidal artery ligation and recto-anal repair in 97 consecutive patients. *Journal of the Korean Society of Coloproctology*.

[28] Faucheron J.-L., Poncet G., Voirin D., Badic B., Gangner Y. (2011). Doppler-guided hemorrhoidal artery ligation and rectoanal repair (HAL-RAR) for the treatment of grade IV hemorrhoids: long-term results in 100 consecutive patients. *Diseases of the Colon and Rectum*.

[29] Gupta P. J., Kalaskar S., Taori S., Heda P. S. (2011). Doppler-guided hemorrhoidal artery ligation does not offer any advantage over suture ligation of grade 3 symptomatic hemorrhoids. *Techniques in Coloproctology*.

[30] Schuurman J.-P., Borel Rinkes I. H., Go P. M. (2012). Hemorrhoidal artery ligation procedure with or without doppler transducer in grade II and III hemorrhoidal disease: a blinded randomized clinical trial. *Annals of Surgery*.

[31] Aigner F., Bodner G., Conrad F., Mbaka G., Kreczy A., Fritsch H. (2004). The superior rectal artery and its branching pattern with regard to its clinical influence on ligation techniques for internal hemorrhoids. *American Journal of Surgery*.

[32] Aigner F., Bodner G., Gruber H. (2006). The vascular nature of hemorrhoids. *Journal of Gastrointestinal Surgery*.

[33] Willis S., Junge K., Ebrahimi R., Prescher A., Schumpelick V. (2010). Haemorrhoids—a collagen disease?. *Colorectal Disease*.

[34] Awojobi O. A. (1983). Modified pile suture in the outpatient treatment of hemorrhoids. A preliminary report. *Diseases of the Colon and Rectum*.

[35] Block I. R. (1985). Obliterative suture technique for internal hemorrhoidectomy. *Diseases of the Colon and Rectum*.

[36] Farag A. E. (1978). Pile suture: a new technique for the treatment of haemorrhoids. *British Journal of Surgery*.

[37] Hussein A. M. (2001). Ligation-anopexy for treatment of advanced hemorrhoidal disease. *Diseases of the Colon and Rectum*.

